# Graphene-Based, Flexible, Wearable Piezoresistive Sensors with High Sensitivity for Tiny Pressure Detection

**DOI:** 10.3390/s25020423

**Published:** 2025-01-13

**Authors:** Rui Li, Jiahao Hu, Yalong Li, Yi Huang, Lin Wang, Mohan Huang, Zhikun Wang, Junlang Chen, Yan Fan, Liang Chen

**Affiliations:** 1 College of Optical, Mechanical and Electrical Engineering, Zhejiang A&F University, Hangzhou 311300, China; lirui1012@stu.zafu.edu.cn (R.L.);; 2 School of Physical Science and Technology, Ningbo University, Ningbo 315211, China

**Keywords:** piezoresistive sensors, graphene oxide, tiny pressure detection, wearable device, copper nanoparticles

## Abstract

Flexible, wearable, piezoresistive sensors have significant potential for applications in wearable electronics and electronic skin fields due to their simple structure and durability. Highly sensitive, flexible, piezoresistive sensors with the ability to monitor laryngeal articulatory vibration supply a new, more comfortable and versatile way to aid communication for people with speech disorders. Here, we present a piezoresistive sensor with a novel microstructure that combines insulating and conductive properties. The microstructure has insulating polystyrene (PS) microspheres sandwiched between a graphene oxide (GO) film and a metallic nanocopper-graphene oxide (n-Cu/GO) film. The piezoresistive performance of the sensor can be modulated by controlling the size of the PS microspheres and doping degree of the copper nanoparticles. The sensor demonstrates a high sensitivity of 232.5 kPa^−1^ in a low-pressure range of 0 to 0.2 kPa, with a fast response of 45 ms and a recovery time of 36 ms, while also exhibiting excellent stability. The piezoresistive performance converts subtle laryngeal articulatory vibration into a stable, regular electrical signal; in addition, there is excellent real-time monitoring capability of human joint movements. This work provides a new idea for the development of wearable electronic devices, healthcare, and other fields.

## 1. Introduction

Flexible, wearable electronics can convert external stresses into electrical signals [1], and therefore, have great potential for applications in areas such as electronic skin [2] and human health monitoring [3,4]. At present, a large number of pressure-sensing mechanisms have been reported, including piezoresistive [5], capacitive [6], piezoelectric [7] and friction electric [8]. Among them, piezoresistive pressure sensors are widely applied for their convenient signal reading [9], simple structure [10] and good stability [11].

Flexible piezoresistive sensors can be widely used in health monitors for they can be attached to the surface of human skin to recognize physiological signals [12,13,14]. Although patients with vocal cord injuries may have hoarseness and even complete loss of voice in severe cases, there are still slight vibrations in the throat joints during vocalization [15,16]. If the different signals of tiny vibrations can be detected through flexible piezoresistive sensors, it will help to improve communication convenience for patients with vocal cord injuries. However, the high-sensitivity pressure range of existing sensors mainly focuses on 10–30 kPa [17,18,19,20,21], while the pressure generated by vocal vibration of the larynx is mainly around 0.3 kPa [22,23]. Therefore, flexible piezoresistive sensors with high sensitivity in this low-pressure range are essential for throat laryngeal articulatory vibration detection.

Microstructure design has become the key approach to improving the sensitivity of piezoresistive sensors, which serves mainly to amplify mechanical loading effects [24]. These microstructures mainly include geometric surfaces, such as pyramids [25]; bionic surfaces, such as flower petals [26], and ginkgo leaves [27]); and object surfaces, such as sandpaper [28], and vinyl record [29]. However, these template-assisted methods often require complex preparation processes and high-cost manufacturing molds. Therefore, constructing microstructures simply and quickly will effectively reduce the preparation cost. In addition, different conductive materials also have an effect on the piezoresistive performance of the sensor [30]. In recent years, various conductive materials have been reported, such as MXene [31,32], carbon nanotubes [33], metal nanowires [34,35] and graphene [36,37,38]. Graphene has been widely used in the preparation of pressure sensors due to its excellent electrical properties, such as its high mobility of charge carriers and high electrical conductivity [39,40,41].

Here, we present a flexible wearable piezoresistive sensor with high sensitivity in a low-pressure range (0–0.2 kPa). The fabrication process is simple and low cost, comprising a microstructure of GO film, n-Cu/GO film and PS microspheres. By dropping PS microsphere solution (8.3 mg/mL) on GO film, after drying, PS microspheres were attached to the GO film. The PS microspheres were sandwiched as microstructures between the graphene oxide membrane and the n-Cu/GO film, which was a fast and low-cost construction process. The prepared sensor, which has the advantages of high sensitivity (232.5 kPa^−1^, 0–0.2 kPa), fast response/recovery time (45 ms/36 ms), and outstanding stability (2800 cycles), can recognize the laryngeal articulatory vibration of different words and the vibration of bending of the joint parts of the human body. These properties arise from the novel microstructure that combines insulating and conductive properties, with insulating PS microspheres sandwiched between a GO film and a metallic nanocopper in graphene oxide film. The present work provides a rapid method for constructing a sensor microstructure, and the sensor with this microstructure has a highly sensitive response at a range of pressure below 10 kPa. This flexible sensor can detect the laryngeal articulatory vibration pressure, which is expected to improve the communication convenience for patients with vocal cord injuries.

## 2. Experimental Section

### 2.1. Materials

The following chemical reagents were used: graphite powder (325 mesh), sulfuric acid (H_2_SO_4_), potassium persulfate (K_2_S_2_O_8_), phosphorus pentoxide (P_2_O_5_), potassium permanganate (KMnO_4_), hydrogen peroxide (H_2_O_2_), hydrochloric acid (HCl), xylene (C_8_H_10_), oleic acid (C_18_H_34_O_2_), oleylamine (C_18_H_37_N), copper acetate (C_4_H_6_CuO_4_), L (+)-ascorbic acid (C_6_H_8_O_6_), and PS microspheres. All of the above chemical reagents were purchased from Shanghai Aladdin Biochemical Technology Co., Ltd. (6F, Sanda Building, No.1 Xinjinqiao Road, Pudong District, Shanghai, China). Analytically pure, deionized water with a resistivity of 18.2 MΩ·cm^−1^ was used for all experiments.

### 2.2. Preparation of n-Cu/GO Solution

A GO suspension was prepared from graphite powder according to the modified Hummers method as previously reported [42,43]. Graphite powder was added to a solution of sulfuric acid, potassium persulfate, and phosphorus pentoxide, and pre-oxidized with continuous stirring for 4.5 h, then washed with deionized water (DI) and centrifuged; pre-oxidized graphite was obtained by vacuum-drying at 60 °C; the graphite was further oxidized using H_2_SO_4_ and KMnO_4_, diluted with DI water, and added to a 30% H_2_O_2_ solution; the resulting product was washed with a 1:10 hydrochloric acid aqueous solution and centrifuged. Deionized water was used to remove impurities from the solution; it was diluted with one liter of deionized water, and then ultrasonicated; finally, a GO suspension was obtained with a concentration of about 5 mg/mL.

Copper nanoparticles were prepared using anhydrous copper acetate according to the reported method of copper nanoparticle preparation [44]. Anhydrous copper acetate, oleic acid and oleylamine were dissolved in xylene and heated to 95 °C in a water bath; then L (+)-ascorbic acid solution (1 mol/L) was added for the reduction; the reaction solution was kept at 95 °C for 3 h, and then cooled to room temperature; finally, the product was collected by centrifugation and washed with acetone and ethanol.

A 5 mL sample of the GO suspension (5 mg/mL) prepared above was added to a small beaker; 4.4 mg of copper nanoparticles were then weighed and added to the GO solution, stirred well with a glass rod, and finally, ultrasonicated for 10 min to complete the preparation of the n-Cu/GO solution.

### 2.3. Preparation of the Sensor

**Figure 1:**
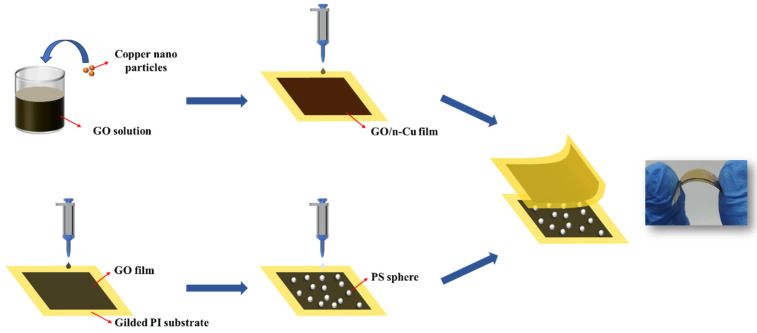
Scheme of the piezoresistive sensor preparation process.

The sensor preparation process is shown in Figure 1. The gold-plated polyimide substrate was fixed, and the GO solution and n-Cu/GO solution were dropped on the gold-plated polyimide substrate using a pipette gun. It was then placed in an oven at 60 °C for drying to form a film. The total amount of dropped solution was 1.5 mL, and to ensure the flatness of the film, small drops were added several times. The film area was 1.5 cm^2^. Then, 60 μL of PS microsphere solution (8.3 mg/mL) was dropped on the GO film, and after drying, copper tape was attached to the gold-plated polyimide substrate. Then, the two membranes were laminated, and finally, encapsulated with a polydimethylsiloxane (PDMS) film to complete the sensor preparation. The PS microspheres are sandwiched between the GO film and the n-Cu/GO film, and the sensor is encapsulated by the PDMS film. This microstructure ensures the stability of the PS microspheres and prevents displacement during testing or practical applications.

### 2.4. Principle Analysis and Characterization

**Figure 2:**
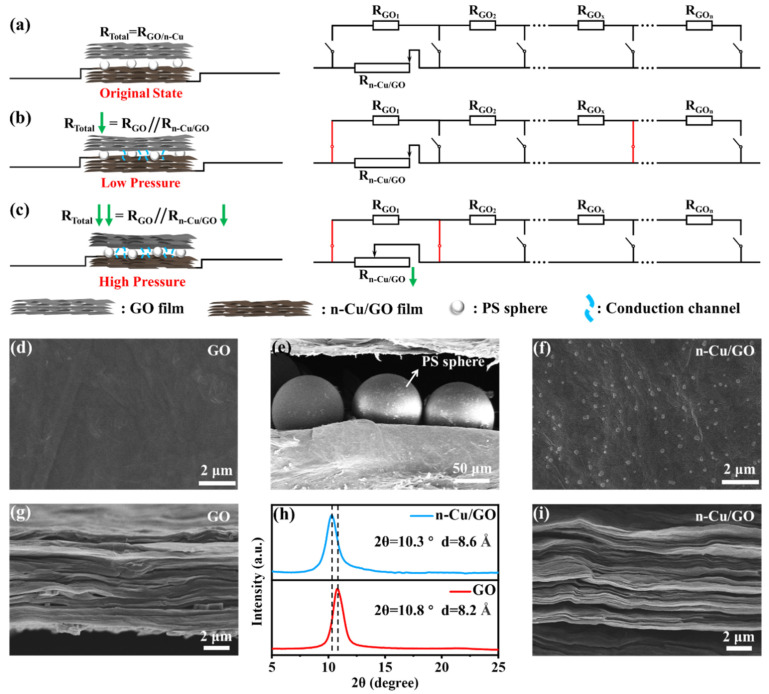
(**a**–**c**) Working principle and corresponding equiv. circuits of the sensor operating at different pressures. (**d**) SEM image of the surface of GO film. (**e**) SEM image of the sandwich structure of the sensor. (**f**) SEM image of the surface of n-Cu/GO film. (**g**) SEM image of cross-section of GO film. (**h**) XRD pattern of GO film and n-Cu/GO film. (**i**) SEM image of cross-section of n-Cu/GO film.

Figure 2a–c demonstrate the piezoresistive sensor’s working principle. When no stress is applied to the sensor, the GO film and the n-Cu/GO film are separated from each other by the sandwiched PS microspheres, and they do not generate a conduction channel. At this time, the sensor is internally connected to the n-Cu/GO film only, and the series circuit is formed internally, with the whole being in a high-resistance state. The sensor is in a high-resistance state, for the PS microspheres are sandwiched between the GO film and the n-Cu/GO film, separating them from each other, as shown in Figure 2a. When the sensor is stimulated by little pressure, the GO film and the n-Cu/GO film contact with each other locally, leading to internal parallel circuits. Then, the total resistance of the sensor decreases, as displayed in Figure 2b. The number of contact points between the two films increases with the increasing pressure on the sensor, which generates more conduction channels, and then the total resistance decreases. Moreover, with large pressure, the internal layer spacing of the GO and n-Cu/GO films decreases, which results in decreases in the film’s own resistance. Then, the total resistance of the sensor decreases further, as demonstrated in Figure 2c. Resistance changes are usually achieved by designing microstructures to change the contact area between the sensitive layer and the electrode [25], or by layering and constructing ion channels or interfaces [45]. In our work, the resistance change is realized by constructing a sandwich structure with a GO film and an n-Cu/GO film separated by the PS microspheres in the middle, and the contact area between the films is changed by applying pressure.

The surface morphology and the cross-section of the sensors were characterized using scanning electron microscopy (SEM). In Figure 2e, the sandwich structure of the sensor is shown clearly, consisting of a GO film, n-Cu/GO film, and PS microspheres. The surface morphology of the GO film and n-Cu/GO film are shown in Figure 2d and Figure 2f, respectively. It can be seen that the roughness of the film surface is increased obviously, and granular bumps are observed after doping with copper nanoparticles. In addition, the cross-sectional views of the GO film and n-Cu/GO film are shown in Figure 2g and Figure 2i, respectively. A clear layer structure can be seen, where the n-Cu/GO film is more sparsely interlayered and the interlayer spacing becomes larger. The film layer spacing was subsequently tested by X-ray diffraction (XRD) experiments and the results are displayed in Figure 2h. After doping with copper nanoparticles, the spacing of the film layers changed by 0.4 Å, which is consistent with the SEM test results. In short, after doping with copper nanoparticles, the surface of GO film is rougher, and the spacing of film layers becomes larger, which causes the change in the film piezoresistive performance. From Figure 2, it can be seen that this sensor creates a microstructure with insulating PS microspheres sandwiched between the GO film and n-Cu/GO film. The conductivity of the GO film is further adjusted by dropping copper nanoparticles to change the interlayer spacing of GO. This novel microstructure that combines insulating and conductive properties leads to the high sensitivity.

### 2.5. Measurement Instruments

SEM (Hitachi Model S-3400N) was used to characterize the sensor structures, film surface morphology and film cross-section structures. XRD (Bruker D8 Advance) was used to measure the film layer spacing. The electrochemical workstation (CHI760e Shanghai Chenhua Instrument Co., Ltd.) was used to test the sensor response time and recovery time. Picoammeter Model 6487 (KEITHLEY, Cleveland, OH, USA) and a press (Mark-10) were used to test the transducers for changes in resistance at different pressures and for stability experiments.

## 3. Results

### 3.1. Piezoresistive Performance

**Figure 3:**
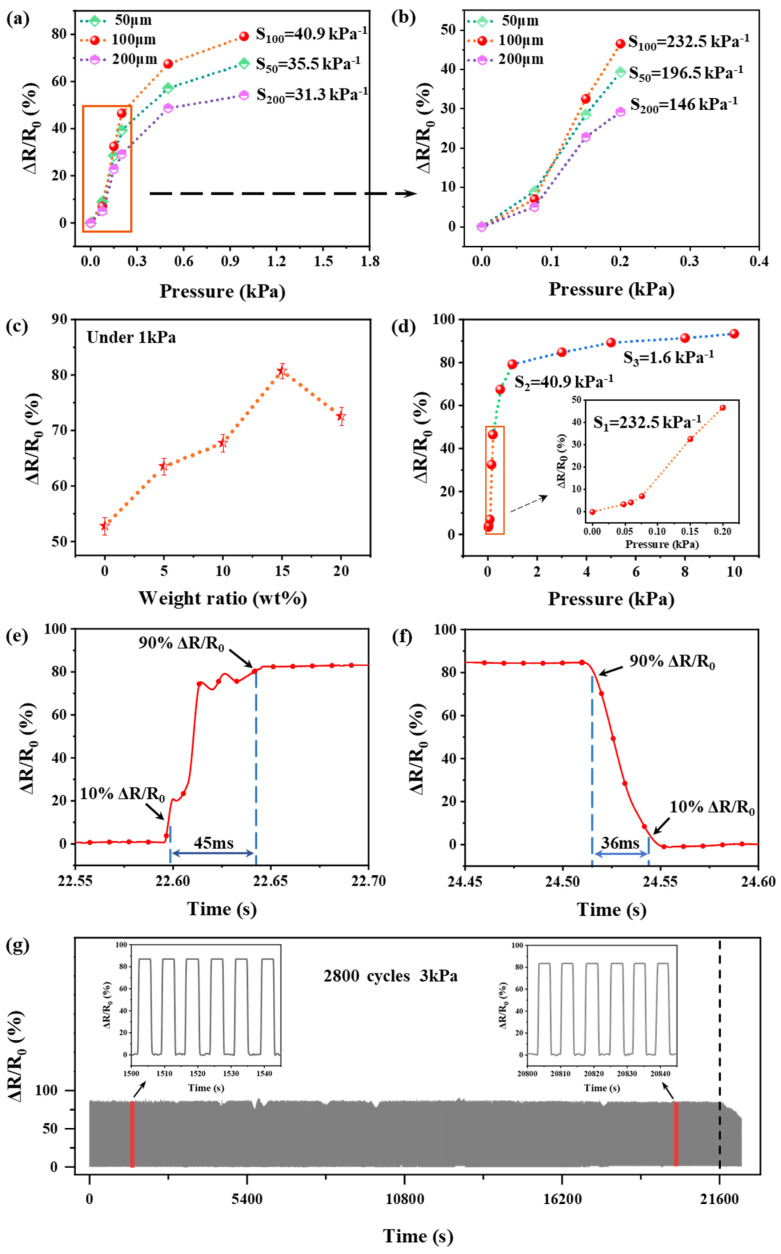
(**a**,**b**) Resistance change rate of sensors prepared of different particle sizes (50 μm, 100 μm and 200 μm); PS microspheres at different pressures. (**c**) Resistance change rate of sensors with copper nanoparticle dopings of 0 wt%, 5 wt%, 10 wt%, 15 wt% and 20 wt% at 1 kPa pressure. (**d**) Resistance change rate versus the pressure applied to the sensor. (**e**,**f**) Response time and recovery time of the sensor. (**g**) The durability of the sensor for loading and unloading pressure for 2800 cycles.

Due to the sandwich structure discussed above, the PS microsphere particle size and copper nanoparticle doping degree played an important role in the piezoresistive performance of the sensor and should be explored to achieve optimal performance of the sensor. During the preparation of the sensors, PS microspheres with different particle sizes (50 μm, 100 μm and 200 μm) were dropped with a solution concentration of 8.3 mg/mL to prepare three different samples. The aim was to investigate the effect of PS microsphere particle size on the piezoresistive performance of the sensors, and the experimental results are shown in Figure 3a,b. The sensor with 100 μm PS microspheres had the highest sensitivity (232.5 kPa^−1^ and 40.9 kPa^−1^) in the pressure intervals of 0–0.2 kPa and 0.2–1 kPa, respectively. The sensitivity is calculated as S = (ΔR/R_0_)/ΔP, where S is the sensitivity, (ΔR/R_0_) is the rate of change in resistance in percent, and ΔP is the change in pressure. It is demonstrated that the particle size of the PS microspheres is too small to separate the GO and n-Cu/GO films effectively, which leads to a connection of these two films just when small pressure stimulates the sensor. On the other hand, a sensor with large particle size PS microspheres requires greater pressure to bring the GO and n-Cu/GO films into contact and create conduction channels, which is not conducive to the sensor’s detection of small pressure. In order to investigate the effect of copper nanoparticle doping degree on the piezoresistive performance of the sensor, samples with five doping levels (0 wt%, 5 wt%, 10 wt%, 15 wt%, and 20 wt%) were prepared and the rates of change of the resistance were tested at a pressure of 1 kPa; the results are displayed in Figure 3c. The sensor sample with a doping degree of 15 wt% has the largest resistance change rate of about 80% at 1 kPa pressure. With doping degrees lower than 15 wt%, the change in film layer spacing is not obvious, and the enhancement of film deformation ability is limited. When the doping degree exceeds 15 wt%, the hardness of the film increases, and the deformation is difficult. Thus, the sensors used in the following tests were coated with 100 μm particle size PS microspheres, and the solution doping of copper nanoparticles was 15 wt%.

To further investigate the piezoresistive performance of the sensors, Figure 3d shows the relationship between the rate of change in resistance at different pressures for the sensors prepared by dropping a solution of PS microspheres with a particle size of 100 μm and doping with copper nanoparticles at a doping amount of 15 wt%. Sensitivity was calculated by dividing the rate-of-change-of-resistance–pressure curve into three linear parts. The sensor has high sensitivity in the low-pressure range of 0–0.2 kPa, with a sensitivity of 232.5 kPa^−1^; in the range of 0.2–1 kPa, it has a sensitivity of 40.9 kPa^−1^; and in the range of 1–10 kPa, it has a sensitivity of 1.6 kPa^−1^. The decrease in sensitivity in the high-pressure range is mainly due to the fact that the contact sites between the GO and n-Cu/GO films and the film’s own morphology gradually approach saturation. Moreover, the response time and recovery time of this sensor were 45 ms and 36 ms, respectively, under the applied 1 kPa pressure, as shown in Figure 3e,f. The sensor also showed good stability in long-term operation, as shown in Figure 3g, where the sensor has a stable continuous pressure release process after 2800 times at a pressure of 3 kPa.

In summary, the sensors prepared in this work have high sensitivity in the low-pressure region (0–0.2 kPa, 232.5 kPa^−1^), which is suitable for detecting small pressures, such as laryngeal articulatory vibration. Compared to the existing related sensors already reported, the present sensor has advantages in terms of response time, recovery time and stability, as shown in Table 1.

### 3.2. Applications

**Figure 4:**
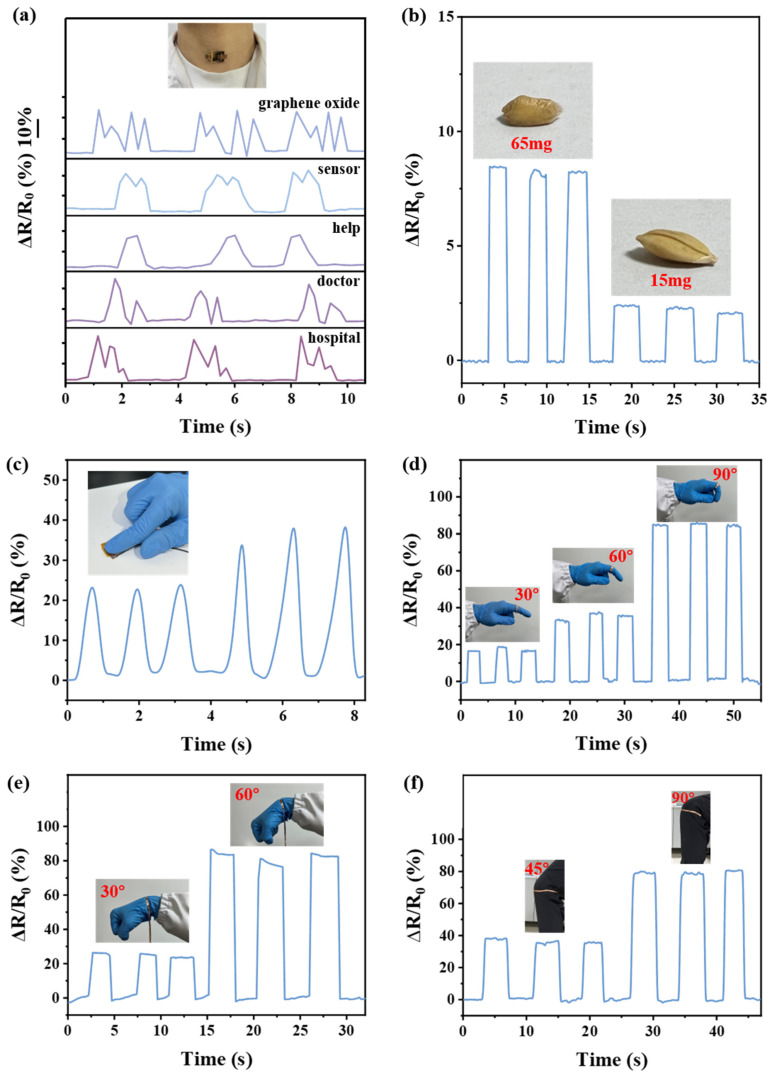
(**a**) Signal responses caused by laryngeal articulatory vibration when the sensor was attached to the larynx. (**b**) Electrical signals of the sensor caused by wheat and rice. (**c**) Electrical signals generated by periodic finger presses. (**d**) Electrical signals generated by bending finger at different angles. (**e**) Electrical signals generated by bending wrist at different angles. (**f**) Electrical signals generated by bending knee at different angles.

Our sensor is characterized by low voltage, high sensitivity, good stability and a fast response time, resulting in broad application prospects in the field of human body monitoring. Since the high-sensitivity pressure range of the sensor matches better with the pressure range generated by laryngeal articulatory vibration, we mounted the sensor on the larynx of human volunteer for real-time monitoring of laryngeal articulatory vibration. As shown in Figure 4a, the sensor is attached to the larynx of the volunteer, who pronounces the words “graphene oxide”, “sensor”, “help”, “doctor” and “hospitals” normally. The volunteer’s larynx produces small vibrations due to phonation, and the sensor can convert the small vibrations into electrical signals in real time, which are monitored and recorded in real time by the Model 6374 Pianograph (KEITHLEY, Cleveland, OH, USA). It can well recognize and distinguish words such as “help”, “doctor” and “hospitals”, which would be sufficient for a basic call for help for patients with speech disorders. The sensor recognizes and spells a wide range of words including monosyllabic words, multi-syllabic words and phrases. In addition, the sensor characterizes the peaks of human articulation with a high degree of differentiation. The sensor prepared in this work has the potential to be used as a wearable artificial throat to improve the ease of communication for patients with speech disorders. Based on the sensor’s outstanding ability to detect small stresses, it can also be used to detect the response of small objects, as shown in Figure 4b. Objects of tiny mass common in life, such as wheat and rice grains, are placed on the sensor, which can clearly distinguish the masses of these two tiny objects.

In addition, the sensors can monitor human movement. The sensors are mounted on the fingers and joint parts of the human body, and when the fingers are lightly touched and the joints are flexed, the sensors can monitor the finger pressing action and the changing trend for the joint parts in real time, as shown in Figure 4c–f. In the experiment, the sensor is lightly touched by the finger, and different electrical signals are generated with different pressures applied by the finger. These signals could be applied to monitor finger grasping movements. When the finger joints, wrists and knees are flexed, the output of the sensor comprises regular, stable and repeatable electric signals. These signals change accordingly when the degree of flexion is different, which demonstrates the reliability of the sensor in monitoring human body-related data.

## 4. Conclusions

In summary, we proposed a novel piezoresistive sensor that employs a new microstructure and has insulating and conductive properties. This sandwich microstructure consists of a graphene oxide (GO) film, an n-Cu/GO film with regulatable conductivity and insulating PS microspheres between them. By optimizing the particle size of the PS microspheres and the doping degree of copper nanoparticles, a highly sensitive sensor in the low-pressure range (0–0.2 kPa, 232.5 kPa^−1^) was obtained. In addition, the prepared sensors have a fast response time and recovery time (45 ms/35 ms), as well as excellent stability (2800 cycles). These excellent piezoresistive properties enable the sensors to monitor human activity, such as laryngeal articulation vibrations and joint flexion movements, in real time. The novel piezoresistive sensors prepared in this work can inject new design concepts for flexible electronic products, which are expected to be applied in practical scenarios such as wearable artificial throats and human motion monitoring.

## Figures and Tables

**Table 1:** Performance comparison of related piezoresistive sensors.

**Sensitivity (kPa^−1^)**	**Response**	**Recovery**	**Stability**	**Ref.**
27.6 (1–1.5 kPa)	800 (μs)	800 (μs)	10,000	[46]
7.023 (0–0.1 kPa)	60 (ms)	80 (ms)	80,000	[47]
1.0005 (0–0.1 kPa)	40 (ms)	40 (ms)	500	[48]
13.89 (0–6 kPa)	64 (ms)	165 (ms)	500	[49]
0.42 (0−1.5 kPa)	36 (ms)	36 (ms)	10,000	[50]
45.81 (0–0.5 kPa)	18 (ms)	36 (ms)	5000	[51]
0.33 (0–1.9 kPa)	72 (ms)	99 (ms)	5000	[52]
132 (0–5.7 MPa)	---	---	10,000	[53]
2.65 (0–0.3 kPa)	80 (ms)	80 (ms)	5000	[25]
20.33	186 (ms)	242 (ms)	9000	[26]
403.46 (10–18 kPa)	105.3 (ms)	99.3 (ms)	12,000	[27]
0.24 (0–2.2 kPa)	15 (ms)	20 (ms)	7000	[29]
0.144 (0–1 kPa)	150 (ms)	150 (ms)	1000	[28]
232.5 (0–0.2 kPa)	45 (ms)	36 (ms)	2800	This work

## Data Availability

If raw data is needed, it can be provided via email.
